# Editorial: Not so WEIRD after all? Relationship science in diverse samples and contexts

**DOI:** 10.3389/fpsyg.2023.1162324

**Published:** 2023-03-07

**Authors:** Veronica M. Lamarche, Kenneth Tan, Sarah C. E. Stanton, Kathleen L. Carswell

**Affiliations:** ^1^Department of Psychology, University of Essex, Colchester, United Kingdom; ^2^School of Social Sciences, Singapore Management University, Singapore, Singapore; ^3^School of Philosophy, Psychology and Language Sciences, University of Edinburgh, Edinburgh, United Kingdom; ^4^Department of Psychology, Durham University, Durham, United Kingdom

**Keywords:** WEIRD, relationship science, single, asexuality, interracial relationships, low-income families

## Introduction

Like many areas of psychology, relationship science suffers from historically drawing from “WEIRD” samples and stimuli (i.e., Western, educated, industrialized, rich, democratic; Karney and Bradbury, 1995; Henrich et al., 2010a,b; Judd et al., 2012). These biases are compounded through an over-representation of people who identify as straight, monogamous, and are interested in long-term partnerships (vs. being single) (Ogolsky and Stafford, 2022; Pollitt et al., 2022; Williamson et al., 2022). Despite over a decade's worth of awareness of the lack of inclusivity, relationship science remains largely dependent on biased samples (IJzerman et al., 2021; Ogolsky and Stafford, 2022; Williamson et al., 2022). Part of the difficulty in addressing the lack of diversity in the literature lies in barriers to publishing work from diverse populations and diverse contexts in top journals in the field. However, if relationship science does not strive to become more inclusive with the samples and contexts in which theoretical advancements are examined, then our understanding of important relationship processes will not advance.

To increase the credibility of relationship science, traditional barriers to publishing research employing diverse samples and contexts must be removed (Maner, 2014; Nosek and Lakens, 2014). This includes barriers associated with treating diversity as a niche or specialist topic, separate from developing generalizable theoretical models. Additionally, the inclusion and extension of research to diverse samples needs to be thoughtful to ensure that unique insights gained by including diverse populations are not washed out (Allmark, 2004). To support change in the field, we invited authors to submit papers that address relationship processes using a diverse lens. For this call, “diversity” was broadly construed, including—but not limited to—race, ethnicity, culture and religion, sexual orientation (i.e., LGBTQA+), gender minority groups, relationship style (i.e., polyamory, consensual non-monogamy), socioeconomic status, single adults, as well as other underrepresented or marginalized groups not listed above. This call led to four articles which concretely advance our understanding of relationships in diverse populations and contexts. We follow by summarizing two themes which emerged across the papers, as well as each paper's contribution, and conclude with considerations for the field.

## Putting the “person” first: Person-centered vs. variable-centered approaches

One theme that emerged in this Research Topic involved the importance of taking person-centered approaches to understanding relational phenomena. Person-centered approaches assume that a population is made up of subpopulations with shared characteristics (Bergman and Magnusson, 1997; Howard and Hoffman, 2018). Variable-centered approaches, by contrast, emphasize the associations between variables by averaging *across* individuals, which can unintentionally obscure important differences that exist across the population. Person-centered approaches by definition allow for a more holistic account of how different domains within the population contribute to outcomes of interest. Two papers in this issue illustrated the benefits of person-centered approaches for understanding relationship phenomena.

First, Brooks and Morrison applied a person-centered approach to the study of interracial couples and the ways in which multidimensional understandings of race and racism at the institutional, interpersonal, and intrapersonal level can inform subjective experiences within interracial relationships. They found that people with a more nuanced understanding of institutionalized racism and more positive intergroup attitudes (i.e., multiculturalists) were more likely to discuss race and racism with their partner, and reported greater relationship satisfaction. The opposite was true for people with poorer understandings of institutionalized racism and more negative intergroup attitudes (i.e., color-blind types). Furthermore, the different experiences with stigma that subpopulations within multiculturalists and color-blind types had explained different relationship outcomes across profiles.

Similarly, Walsh et al. used a person-centered approach to illustrate how being single is associated with life satisfaction. They identified not only subpopulations wherein being single was more likely to be associated with lower life satisfaction, but also highlighted profiles wherein being single was associated with being happier, particularly for people with more positive personality traits. Taking person-centered approaches to understanding particular phenomena may therefore prove particularly fruitful when considering populations which have historically been excluded from the narrative (e.g., singles, interracial couples), and whose voices would be at risk of being subsumed into aggregate experiences.

## A theory for everyone? Extending theoretical models to underrepresented populations

An aim of psychological research is to identify truths about *human* behavior. However, focusing on restricted populations and assuming these theoretical models apply ubiquitously can provide a false-consensus, and prevent the advancement of theoretical models. Two papers in this issue illustrated the limits of our theories by applying them to populations historically excluded from relationship research.

First, Brozowski et al. applied the investment model (Rusbult et al., 1998) to asexual individuals. Asexuality offers a unique test of the investment model because the ways in which asexual people initiate and maintain their romantic partnerships are often very different compared to allosexual people (e.g., Scherrer, 2010; Robbins et al., 2016). Despite these differences, Brozowski et al.'s study found that satisfaction, investment, and quality of alternatives were antecedents of commitment among their sample of asexual participants, replicating past work with this model. However, they also found that anxious attachment *strengthened* the associations between investment model characteristics and commitment in this sample of asexual participants, rather than weakened them as observed in samples where allosexuality is assumed.

Next, Ross et al. tested the Vulnerability-Stress-Adaptation Model (VSA; Karney and Bradbury, 1995) among couples from lower socioeconomic backgrounds. However, the assumption that this model generalizes across economic levels as predictors of satisfaction remained untested. Despite demonstrating that these were reliable predictors of satisfaction across newlyweds from different socioeconomic backgrounds, they also noted that communication did not mediate the association between vulnerability, stress, and its ensuing effects on satisfaction. These findings cast doubt on a central component of the VSA model. Thus, both papers highlight that while theoretical models may replicate across specific populations, the mechanisms through which they operate may nonetheless be different compared to aggregate samples.

## The challenge of inclusive research and future directions

This Research Topic highlights the value of including underrepresented populations in research for the field. However, several challenges became apparent. First, although each paper included a historically underrepresented population or context, the authorship teams were all based in the United States. Despite greater inclusion over time, the US remains overrepresented in scientific research (Thalmayer et al., 2021), while researchers from the Global South remain underrepresented (Macleod and Howell, 2013; IJzerman et al., 2021; Bernardo et al., 2022; Hattery et al., 2022; Lin and Li, 2022). This may partly be due to the relative marginalization of subdisciplines in some regions outside the US and Western Europe (e.g., social psychology; Saab et al., 2020). There is therefore an important opportunity and urgency for greater collaboration between scholars in the Global North and those in the Global South to advance psychological theories 2-fold: by increasing the visibility of disciplines through collaborative work, and the progression of scientific theory through more inclusive investigations of phenomena.

Finally, concerns about whether a study simply demonstrates what is “already known”, or “is only one failed replication of an entire body of work”, continue to act as barriers, preventing replication and extension in underrepresented contexts out of concern that these investigations are insufficiently novel or aberrantly non-replicable. Relationship science must continue to challenge the implicit assumption that findings from WEIRD/heterosexual/monogamous samples in the Global North/West reflect *known* phenomena, and not a pattern unique to this subgroup of participants, to advance a relationship science that represents the global human experience.

## Conclusion

We embarked on this editorial journey because we believe that relationship science can only be improved by extending our research to historically underrepresented people and contexts. We hope to highlight that, moving forward, researchers will have to challenge systemic biases, as well as barriers in the publication pipeline to ensure the future of relationship science is inclusive and more representative of relationships around the world.

## Author contributions

VL wrote the first draft of this editorial. KT, SS, and KC provided critical feedback and helped shape the final piece. All authors conceived of this editorial and handled a paper in this Research Topic. All authors contributed to editorial revision, read, and approved the submitted version.
